# Low-Density Lipoprotein Electronegativity Is a Novel Cardiometabolic Risk Factor

**DOI:** 10.1371/journal.pone.0107340

**Published:** 2014-09-09

**Authors:** Jing-Fang Hsu, Tzu-Chieh Chou, Jonathan Lu, Shu-Hua Chen, Fang-Yu Chen, Ching-Chu Chen, Jeffrey L. Chen, MacArthur Elayda, Christie M. Ballantyne, Steven Shayani, Chu-Huang Chen

**Affiliations:** 1 L5 Research Center, China Medical University Hospital, Taichung, Taiwan; 2 Department of Public Health, China Medical University, Taichung, Taiwan; 3 Department of Health Risk Management, China Medical University, Taichung, Taiwan; 4 Vascular and Medicinal Research, Texas Heart Institute, Houston, Texas, United States of America; 5 Department of Internal Medicine, China Medical University Hospital, Taichung, Taiwan; 6 Physical Medicine & Rehabilitation, Department of Orthopedic Surgery, University of California San Diego, San Diego, California, United States of America; 7 Biostatistics and Epidemiology, Texas Heart Institute, Houston, Texas, United States of America; 8 Department of Medicine, Baylor College of Medicine, Houston, Texas, United States of America; 9 Mount Sinai Medical Center, New York, New York, United States of America; 10 New York Heart Research Foundation, New York, New York, United States of America; 11 Department of Medicine, Kaohsiung Medical University, Kaohsiung, Taiwan; Innsbruck Medical University, Austria

## Abstract

**Background:**

Low-density lipoprotein (LDL) plays a central role in cardiovascular disease (CVD) development. In LDL chromatographically resolved according to charge, the most electronegative subfraction–L5–is the only subfraction that induces atherogenic responses in cultured vascular cells. Furthermore, increasing evidence has shown that plasma L5 levels are elevated in individuals with high cardiovascular risk. We hypothesized that LDL electronegativity is a novel index for predicting CVD.

**Methods:**

In 30 asymptomatic individuals with metabolic syndrome (MetS) and 27 healthy control subjects, we examined correlations between plasma L5 levels and the number of MetS criteria fulfilled, CVD risk factors, and CVD risk according to the Framingham risk score.

**Results:**

L5 levels were significantly higher in MetS subjects than in control subjects (21.9±18.7 mg/dL vs. 11.2±10.7 mg/dL, *P*:0.01). The Jonckheere trend test revealed that the percent L5 of total LDL (L5%) and L5 concentration increased with the number of MetS criteria (*P*<0.001). L5% correlated with classic CVD risk factors, including waist circumference, body mass index, waist-to-height ratio, smoking status, blood pressure, and levels of fasting plasma glucose, triglyceride, and high-density lipoprotein. Stepwise regression analysis revealed that fasting plasma glucose level and body mass index contributed to 28% of L5% variance. The L5 concentration was associated with CVD risk and contributed to 11% of 30-year general CVD risk variance when controlling the variance of waist circumference.

**Conclusion:**

Our findings show that LDL electronegativity was associated with multiple CVD risk factors and CVD risk, suggesting that the LDL electronegativity index may have the potential to be a novel index for predicting CVD. Large-scale clinical trials are warranted to test the reliability of this hypothesis and the clinical importance of the LDL electronegativity index.

## Introduction

Cardiovascular disease (CVD) is a significant public health problem and is a source of economic burden in the United States and globally [1], [2]. The identification of major CVD risk factors is important for preventing, controlling, and treating this disease. Classic CVD risk factors include hypertension, dyslipidemia, diabetes mellitus, obesity, decreased physical exercise, and smoking [3]–[5]. Among these, the most evident causal disorder for CVD is dyslipidemia, or hypercholesterolemia [6], [7].

Lipids, particularly low-density lipoprotein (LDL), are known to play a central role in CVD development. Avogaro and colleagues [8] were the first to report that LDL could be divided into electropositive [LDL (+)] and electronegative [LDL (–)] fractions by using ion-exchange chromatography. Since then, others have described the chemical and functional properties of these dichotomized LDL subfractions [9]–[12]. The proportion of plasma LDL(–) has been shown to be increased in patients with high cardiovascular risks [13] such as hyperlipidemia, diabetes, severe renal disease, and nonalcoholic steatohepatitis, as well as in patients with coronary syndromes when compared with healthy individuals. Thus, it has been suggested [13] that LDL(–) may be a useful biomarker for cardiovascular risks, but large-scale clinical trials are needed to test the reliability of this hypothesis. We have previously separated human plasma LDL according to charge into 5 subfractions (L1–L5) with increasing electronegativity by using anion-exchange chromatography [14]. Notably, plasma levels of the most electronegative LDL subfraction, L5, are moderately increased in patients with high cardiovascular risks such as hypercholesterolemia, type 2 diabetes mellitus, and smoking [14]–[16], as well as in patients with ST-segment elevation myocardial infarction, when compared with healthy individuals [17], [18]. L5 has a chemical composition unique from that of other LDL subfractions, marked by decreased levels of apolipoprotein B and increased levels of other apolipoproteins [19]. In addition, L5 is not internalized by the normal LDL receptor but by the lectin-like oxidized LDL receptor- 1 (LOX-1), which in turn leads to endothelial cell apoptosis [20]. Circulating L5 has been shown to be pro-atherogenic [9] and is the only subfraction of human LDL that induces endothelial dysfunction and atherogenic responses in cultured vascular cells [14], [15], [21]. Although L5 has a role in promoting atherogenesis, it remains unknown whether L5 levels increase with the progression of CVD. Understanding the association between L5 and the progression of CVD may help clinicians counsel patients on lifestyle interventions while fueling research to identify preventions or interventions for CVD.

MetS is a clustering of metabolic abnormalities that are collectively associated with an increased risk of coronary heart disease, stroke, diabetes mellitus, CVD events, and cardiovascular mortality [22]–[25]. The combined presence of these abnormalities poses a greater risk than the presence of each component alone, which makes MetS useful for identifying high-risk individuals. Recently, we have shown that plasma L5 levels are elevated in individuals with MetS [26], suggesting that L5 levels may be associated with CVD progression and could be a novel CVD predictor. In this study, we examined plasma L5 levels in asymptomatic individuals with or without MetS and evaluated LDL electronegativity as an index for predicting CVD in these individuals by examining correlations between plasma L5 levels and the number of MetS criteria fulfilled, CVD risk factors, and CVD risk according to the Framingham risk score.

## Methods

### Study subjects

Between 2010 and 2012, we enrolled 57 asymptomatic individuals who had not received statin treatment or any other lipid-lowering therapy in the previous 3 months [27]. Patients who met the criteria for MetS, according to the 2009 Joint Interim Statement of the International Diabetes Federation Task Force on Epidemiology and Prevention (IDF), the National Heart, Lung, and Blood Institute (NHLBI), the American Heart Association (AHA), the World Heart Federation, the International Atherosclerosis Society, and the International Association for the Study of Obesity [28], composed the MetS group, and all other patients were healthy control subjects. All study subjects were seen by cardiologists at the Texas Heart Institute (Houston, Texas) or at the New York Heart Research Foundation (New York, New York). All participants provided written consent for the use of their plasma; the study protocol was approved by the medical ethics committee of Baylor College of Medicine (Houston, Texas) and the New York Heart Research Foundation. The study was conducted according to the principles in the Declaration of Helsinki.

### Sample collection

All subjects were instructed to fast before blood sample collection so that lipid concentrations would represent stable lipid levels. Venous blood samples (30 mL) were drawn from each subject by using BD VACUETTE EDTA Blood Tubes (Becton, Dickinson and Company, UK) containing anti-coagulants. A questionnaire was administered to each subject that included a range of questions relating to personal characteristics (eg, age, sex) and lifestyle (eg, tobacco usage, medical history). Information obtained from the questionnaire was used in the statistical analysis. For all study subjects, analysis of lipid levels and other biochemical parameters was performed in the Department of Laboratory Medicine at the Texas Heart Institute or at the New York Heart Research Foundation (accredited by the College of American Pathologists) according to standard operating procedures.

### LDL isolation and separation of LDL subfractions

LDL (density = 1.063–1.019 g/cm^3^) was isolated from whole blood samples (20 mL) by using sequential potassium bromide density centrifugation and was treated with 5 mmol/L EDTA and nitrogen to avoid ex vivo oxidation [29]. Isolated LDL was separated into 5 subfractions (L1, L2, L3, L4, and L5) with increasing negative charge on anion-exchange columns (Uno-Q12, BioRad, Hercules, CA) by using the ÄKTA fast protein liquid chromatography (FPLC) system (GE Healthcare Life Sciences, Pittsburgh, PA) as described previously [14], [15]. The columns were equilibrated with buffer A (0.02 mol/L Tris–HCl, pH 8.0; 0.5 mmol/L EDTA). Subfractions were eluted with a multistep linear gradient of buffer B (1 mol/L NaCl in buffer A) at a flow rate of 2 mL/min and were monitored at 280 nm. LDL subfractions from subjects were individually concentrated by using Centriprep filters (YM-30; EMD Millipore Corp., Billerica, MA) and were sterilized by passage through 0.22-µm filters. Protein concentrations of LDL subfractions were measured by using the Lowry method [30].

### Cell culture and cell apoptosis assays

Primary bovine aortic endothelial cells (BAECs; Cambrex, East Rutherford, NJ) were used after 3 or 4 passages and were maintained in Dulbecco’s modified Eagle’s medium (DMEM; Invitrogen, Carslbad, CA) containing 10% fetal bovine serum and antibiotics [14]. Subconfluent cultures washed and maintained in DMEM containing 5% serum were exposed for 24 hours to LDL subfractions (50 µg/mL each of L1, L3, or L5) from MetS or non-MetS control subjects. Generally, the percentage of L5 detected in LDL from healthy individuals is very low. Therefore, all of the LDL subfractions (L1, L3, or L5) were pooled from several MetS or non-MetS subjects to obtain the amount necessary for cell experiments. Treated cells were stained for 10 minutes with 1 mol/L Hoechst 33342 (Molecular Probes, Grand Island, NY) to assess nuclear morphology and with calcein acetoxymethyl ester and propidium iodide (Molecular Probes) to assess membrane integrity. Epifluorescence imaging (500 cells/well) was performed in triplicate by using a Zeiss inverted microscope (Axiovert; x400) with MetaView software (Universal Imaging Corp., Downington, PA).

### CVD risk

Three CVD risks were calculated by using the Framingham risk score (developed by the large epidemiological Framingham Heart Study): the 10-year risk of “general” CVD [31], the 30-year risk of “general” CVD, and the 30-year risk of “hard” CVD [32]. The “general” CVD category included coronary death, myocardial infarction, coronary insufficiency, angina, ischemic stroke, hemorrhagic stroke, transient ischemic attack, peripheral artery disease, and heart failure. The “hard” CVD category included coronary death, myocardial infarction, and stroke. The predictors used to calculate the Framingham risk score included sex, age, blood pressure, information regarding the treatment of hypertension and diabetes mellitus, smoking status, body mass index (BMI), and levels of total cholesterol and high-density lipoprotein (HDL).

### Statistical analysis

All data are presented as frequencies for discrete responses and as the mean ± standard deviation for continuous responses. For all parameters examined in this study, the Shapiro-Wilk normality test was used to determine whether a random sample of values followed a normal distribution. To compare differences between 2 groups, a nonparametric Mann-Whitney test was used for continuous data, and a chi square test or Fisher exact test was used for binary data. The Jonckheere trend test, a nonparametric test specifically designed to detect differences arising from ordered treatments [33], was used to compare the differences in the percent L5 of total LDL (L5%) and the L5 concentration ([L5]; L5% multiplied by LDL concentration) between 6 groups of subjects defined according to the number of MetS criteria met. More formally, the test considered the null hypothesis that, for 6 groups where L5_i_ is an L5 level of the central tendency of the *i*th group, L5_0_ = L5_1_ = L5_2_ = L5_3_ = L5_4_ = L5_5_, against the alternative hypothesis that L5_0_<L5_1_<L5_2_<L5_3_<L5_4_<L5_5_. If the results of the test were statistically significant (*P:* <0.05), the human plasma L5 level was believed to be associated with the progression of metabolic derangement in healthy individuals. The Jonckheere trend test was also used to compare the differences in the concentration of LDL between 6 groups of subjects defined according to the number of MetS criteria met. The associations between L5% and other CVD risk factors, including waist circumference, systolic blood pressure (SBP), diastolic blood pressure (DBP), and levels of fasting plasma glucose, triglyceride, and HDL, were evaluated by using the Spearman rank correlation coefficient, a linear regression model, and a stepwise multiple regression model. Additionally, the association between L5% and CVD risk, as derived by the Framingham risk score [31], [32], was evaluated by using the Spearman rank correlation coefficient and a stepwise multiple regression model. A *P*-value <0.05 was considered statistically significant. Statistical analysis was performed by using the Statistical Package for Social Science (version 19.0; SPSS Inc., Chicago, IL) software system.

## Results

### Clinical characteristics and biochemical parameters of study subjects

Of the 57 subjects enrolled in our study, 19 (33%) were men. The mean age of study subjects was 52.3±10.3 years (range, 32–86 years). Among the study subjects, 38/57 (66.7%) had a waist circumference above normal (≥88 cm for women and ≥102 cm for men), 26/57 (45.6%) had hypertriglyceridemia (triglyceride ≥150 mg/dL), 22/57 (38.6%) had low HDL levels (<50 mg/dL for women and <40 mg/dL for men), 28/57 (49.1% ) had elevated SBP and/or DBP (SBP/DBP, ≥130/85 mmHg), and 22/57 (38.6%) had elevated fasting plasma glucose levels (≥100 mg/dL). Thirty study subjects (52.6%) were determined to have MetS; the remaining 27 subjects who did not meet the criteria for MetS were the healthy control subjects.

### L5 levels in MetS and control subjects

**Figure 1:**
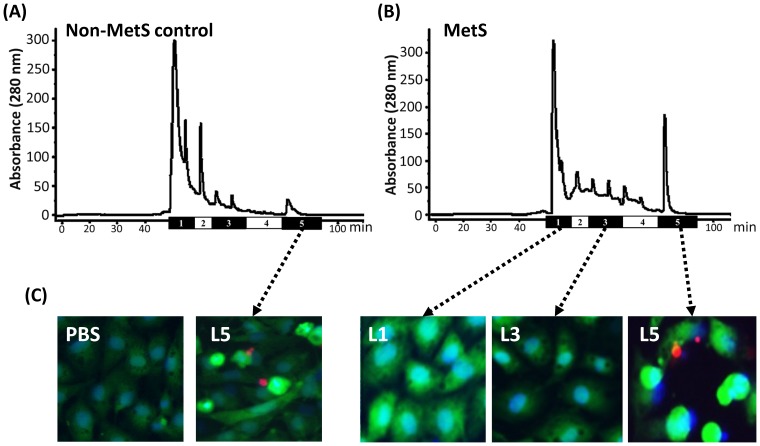
Distribution of LDL subfractions in metabolic syndrome (MetS) and healthy control subjects and the effects of LDL subfractions from MetS subjects on cell death. Representative chromatographs showing the distribution of LDL subfractions L1–L5 (labeled 1–5) in LDL from a (A) control subject and (B) MetS subject. (C) Effects of L1, L3, and L5 (50 µg/mL each) from MetS subjects and L5 (50 µg/mL) from non-MetS control subjects on bovine aortic endothelial cell (BAEC) death after 24 hours, as determined by staining with Hoechst 33342 (to assess nuclear morphology, blue) and calcein acetoxymethyl ester and propidium iodide (to assess membrane integrity, red). As a negative control, BAECs were incubated with phosphate-buffered saline (PBS) for 24 hours. BAECs that have condensed, fragmented nuclei were considered to be undergoing cell apoptosis.

**Figure 2:**
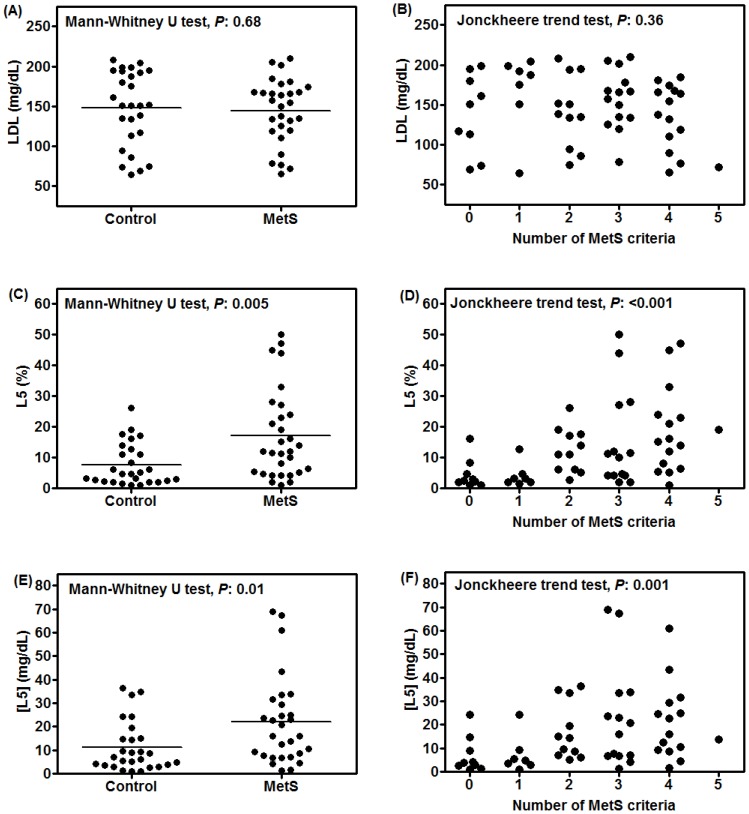
Correlation of low-density lipoprotein (LDL) concentration, L5 percentage (L5%), and L5 concentration ([L5]) with metabolic syndrome (MetS) and the number of MetS criteria. LDL concentration, L5%, and [L5] were plotted for MetS and control subjects and according to MetS criteria. Correlation of LDL concentration with (A) MetS and (B) the number of MetS criteria. Correlation of L5% with (C) MetS and (D) the number of MetS criteria. Correlation of [L5] with (E) MetS and (F) the number of MetS criteria.

In MetS subjects, the distribution of LDL subfractions L1–L5 was shifted more toward the most negatively charged subfractions (ie, L5) when compared with that of control subjects (Figure 1A and 1B). The percentage of L5 in MetS subjects was higher in men than in women (20.0±15.4% vs. 15.2±14.1%, respectively), but the difference was not statistically significant (*P*: 0.38, Mann-Whitney U test). For control subjects, the percentage of L5 was almost the same between men and women (8.0±7.4% vs. 7.4±6.8%, respectively). These findings indicate that among men and women with MetS, the effects of L5 may be more pronounced in men, which is consistent with the previously reported findings of Lee and colleagues [26]. To confirm the biologic effects of LDL subfractions, we examined cell apoptosis in BAECs treated with L1, L3, or L5 from MetS or non-MetS control subjects for 24 hours. L5 from MetS subjects and L5 from non-MetS control subjects each induced a marked increase in cell apoptosis, whereas L1 and L3 had negligible or mild effects, respectively (Figure 1C). In addition, morphological changes were observed in BAECs treated with L5 and L3 but not in those treated with L1. When we compared the 5 interrelated risk factors for MetS (waist circumference, SBP, and levels of fasting plasma glucose, triglyceride, and HDL) between MetS and control subjects, we observed significant differences in all of these parameters (Table 1). Although total cholesterol and LDL levels were not significantly different between these groups (Table 1 and Figure 2A), the L5% and [L5] were significantly higher in MetS subjects than in control subjects (*P:* 0.005 for L5% and *P*: 0.01 for [L5], Figure 2C and 2E). Furthermore, the Jonckheere trend test revealed that L5% and [L5] increased with the number of MetS criteria (*P:* <0.001 for L5% and *P*: 0.001 for [L5], Figure 2D and 2F) but not with LDL level (*P*: 0.36, Figure 2B).

**Table 1:** Characteristics of MetS and healthy control subjects^a^.

	Control	MetS	*P*-value^c^
	n = 27	n = 30	
Gender (men:women)	8∶19	11∶19	0.57
Age (years)	48.8±10.9	55.4±8.7	**0.005**
DM drug treatment	0/27	5/30	**0.05**
Hypertension drug treatment	4/27	12/30	**0.04**
Smoker (no:yes)	13∶14	9∶21	0.16
Waist circumference (cm)	88.4±13.9	107.4±13.5	**<0.001**
Body mass index (kg/m^2^)	27.0±5.9	33.7±6.2	**<0.001**
Waist-to-height (ratio)	0.56±0.08	0.65±0.08	**0.001**
Systolic blood pressure (mmHg)	119.2±20.8	133.8±14.8	**0.001**
Diastolic blood pressure (mmHg)	74.8±9.1	78.9±11.7	0.15
Pulse pressure (mmHg)^b^	44.4±17.3	54.9±14.7	**0.003**
Mean arterial pressure (mmHg)	89.6±11.6	97.2±10.8	**0.03**
Fasting plasma glucose (mg/dL)	90.8±17.2	117.8±45.5	**<0.001**
Total cholesterol (mg/dL)	227.7±60.5	224.4±46.2	0.61
Triglyceride (mg/dL)	118.0±73.9	178.8±108.0	**0.001**
HDL (mg/dL)	60.5±15.4	47.3±12.0	**<0.001**
LDL (mg/dL)	147.9±47.0	144.5±40.6	0.68
L5 (%)	7.6±6.2	17.0±14.5	**0.005**
[L5] (mg/dL)	11.2±10.7	21.9±18.7	**0.01**

a Data are expressed as the mean ± standard deviation or as a ratio.

b Pulse pressure is equal to systolic blood pressure minus diastolic blood pressure.

c The *P*-value was calculated by using the Mann-Whitney U test, excluding sex, hypertension drug treatment, and smoking status variables, which were subjected to the chi square test, and the DM drug treatment variable, which was subjected to the Fisher exact test.

DM, diabetes mellitus; HDL, high-density lipoprotein; LDL, low-density lipoprotein; L5%, percent L5 in total LDL; [L5], concentration of L5; MetS, metabolic syndrome.

### L5 levels and CVD risk factors

**Figure 3:**
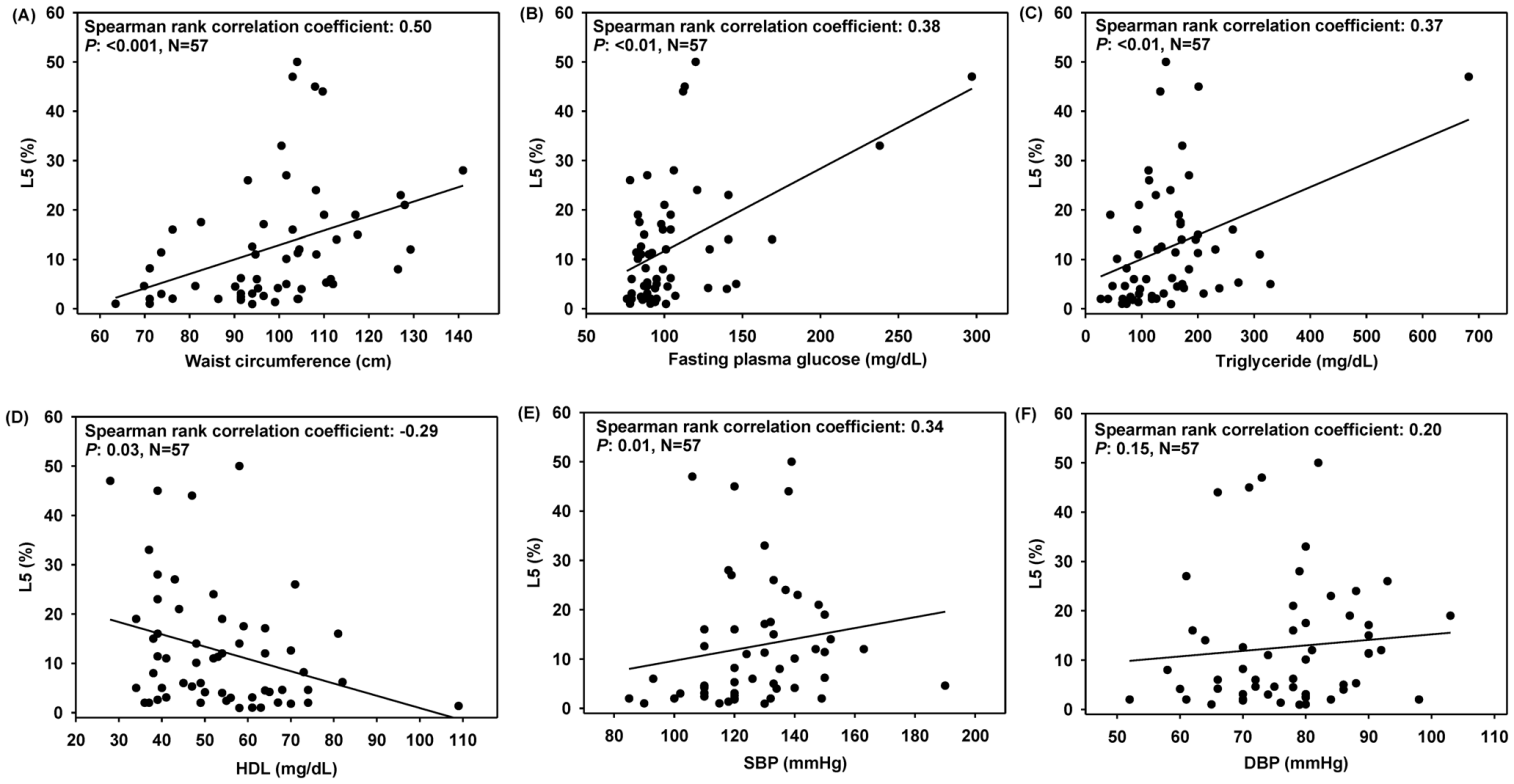
Correlation between L5 percentage (L5%) and various components of metabolic syndrome (MetS) criteria. L5% values were plotted against each component as indicated. Correlation between L5% and (A) waist circumference, (B) fasting plasma glucose, (C) triglyceride, (D) high-density lipoprotein (HDL), (E) systolic blood pressure (SBP), and (F) diastolic blood pressure (DBP).

We evaluated the association between L5 and various CVD risk factors. For all study subjects, L5% increased with increasing waist circumference, SBP, and levels of fasting plasma glucose and triglyceride (Figure 3), as well as BMI, waist-to-height ratio, pulse pressure, and mean arterial pressure (Table 2, *P*: <0.05). HDL level was negatively associated with L5% (*P*: 0.03, Figure 3D). The subjects who were receiving drug treatment for hypertension or who were smokers had a significantly higher L5% than did those who were not receiving treatment or who were not smokers, respectively (Table 3, *P*: <0.05). No statistically significant association was observed between L5% and age, sex, DBP, total cholesterol, or LDL (*P*: >0.05, Tables 2 and 3 and Figure 3F). To evaluate the relationship between L5% and multiple CVD risk factors, we performed stepwise multiple regression analysis. As shown in Table 4, L5% was associated with fasting plasma glucose level and BMI (*P*: <0.05), and these 2 factors contributed to 28% of L5% variance (R^2^∶0.28, *P*: <0.01). The results of multiple regression analysis also revealed that L5% increased by 0.14% for every 1 mg/dL increase in fasting plasma glucose level and 0.58% for every 1 kg/m^2^ increase in BMI (Table 4).

**Table 2:** Correlation of L5% and various CVD risk factors.

	L5 (%)	
	Spearman Rank Correlation Coefficient (rho)	*P*-value
Age (years)	0.08	0.57
Body mass index (kg/m^2^)	0.49	<0.001
Waist-to-height (ratio)	0.29	0.04
Pulse pressure (mmHg)^a^	0.31	0.02
Mean arterial pressure (mmHg)	0.29	0.03
Total cholesterol (mg/dL)	–0.08	0.58
LDL (mg/dL)	–0.08	0.54

a Pulse pressure is equal to systolic blood pressure minus diastolic blood pressure.

CVD, cardiovascular disease; LDL, low-density lipoprotein; L5%, percent of L5 in total LDL.

**Table 3:** Comparison of L5% in subjects grouped according to characteristics.

Characteristic	n	L5 (%)^a^	*P*-value^b^
Sex			0.37
Men	19	15.0±13.8	
Women	38	11.3±11.6	
DM drug treatment			0.26
No	52	11.7±11.5	
Yes	5	20.6±18.8	
Hypertension drug treatment			**0.006**
No	41	10.2±11.3	
Yes	16	18.4±13.4	
Smoker			**0.02**
No	22	6.9±5.2	
Yes	35	16.0±14.2	

a L5 (%) values are expressed as the mean ± standard deviation.

b The *P*-value was calculated by using the Mann-Whitney U test.

DM, diabetes mellitus.

**Table 4:** Multivariate analysis of L5% in terms of fasting plasma glucose level and body mass index (N = 57).

Independent Variable	Coefficients	*P*-value	Model R, *P*-value^a^
			0.53, <0.01
Constant	–20.03	<0.01	
Fasting glucose	0.14	0.02	
Body mass index	0.58	<0.01	

a Stepwise multiple regression analysis was used to select the independent variables in this model. The independent variables included age, sex, smoking status, body mass index, waist circumference, blood pressure, pulse pressure, mean arterial pressure, fasting plasma glucose level, total cholesterol level, triglyceride level, high-density lipoprotein level, and low-density lipoprotein level.

### L5 levels and CVD risk

**Figure 4:**
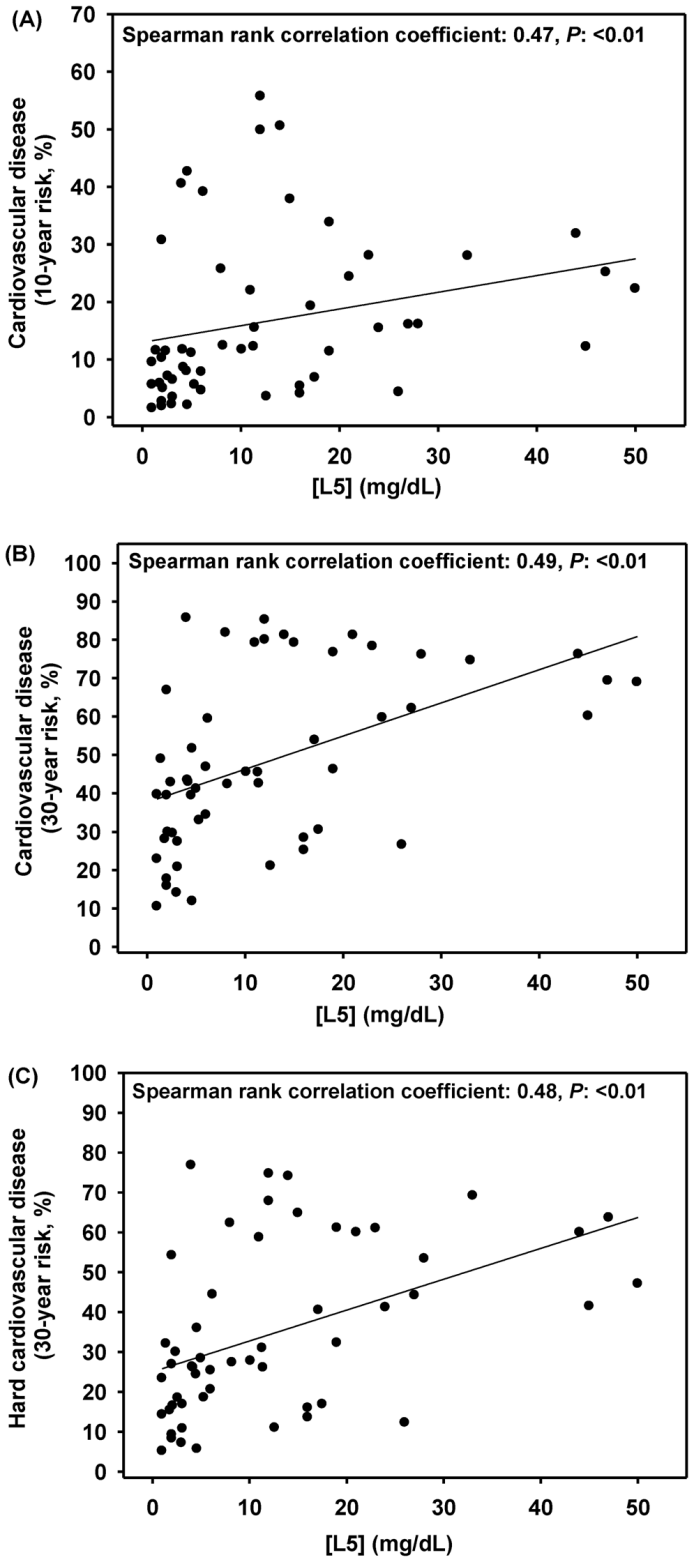
Correlation between L5 concentration ([L5]) and cardiovascular disease (CVD) risks. L5 concentration values are plotted against each risk as indicated. Correlation between [L5] and (A) 10-year general CVD risk, (B) 30-year general CVD risk, and (C) 30-year hard CVD risk.

To further evaluate whether L5 levels have the potential to be a novel CVD predictor, we examined the relationship between [L5] and CVD risks, as calculated by using the Framingham risk score [31], [32]. The 10- and 30-year risks of general CVD were highly correlated with [L5] (Spearman rank correlation coefficient: 0.47 and 0.49, respectively; *P*: <0.01, Figure 4). The 30-year risk of “hard” CVD was also highly correlated with [L5] (Spearman rank correlation coefficient: 0.42, *P*: <0.01). To extract the contribution of [L5] to CVD risk, we performed stepwise multiple regression analysis. As shown in Table 5, CVD risks were associated with [L5] and waist circumference. The [L5] and waist circumference contributed to a total of 23% (R^2^∶0.23, *P*<0.01), 59% (R^2^∶0.59, *P*<0.01), and 52% (R^2^∶0.52, *P*<0.01) of variance for 10-year general CVD risk, 30-year general CVD risk, and 30-year hard CVD risk, respectively. When we determined the contribution of [L5] to CVD risks when controlling the variance of waist circumference, we found that [L5] contributed to 11% of 30-year general CVD risk (partial r^2^∶0.11, *P*: 0.02) and 8% of 30-year hard CVD risk (partial r^2^∶0.08, *P*: 0.04) variances. No statistically significant association was observed between [L5] and 10-year general CVD risk (partial r^2^∶0.02, *P*: 0.28).

**Table 5:** Multivariate analysis of CVD risks in terms of L5 concentration [L5] and waist circumference (N = 57).

	Coefficients	*P*-value	Partial correlation r	Model R, *P*-value^a^
**Dependent variable: 10-year risk of cardiovascular disease**				**0.48, <0.01**
Independent variable:				
Constant	–19.43	0.07		
[L5]	0.12	0.28	0.15	
Waist circumference	0.34	<0.01	0.40	
**Dependent variable: 30-year risk of cardiovascular disease**				**0.77, <0.01**
Independent variable:				
Constant	–44.04	<0.01		
[L5]	0.32	0.02	0.33	
Waist circumference	0.88	<0.01	0.70	
**Dependent variable: 30-year risk of hard cardiovascular disease**				**0.72, <0.01**
Independent variable:				
Constant	–45.91	<0.01		
[L5]	0.28	0.04	0.29	
Waist circumference	0.77	<0.01	0.65	

a Stepwise multiple regression analysis was used to select the independent variables used in this model. The independent variables included the plasma concentration of L5 ([L5]), waist circumference, diastolic blood pressure, total cholesterol level, triglyceride level, high-density lipoprotein level, and low-density lipoprotein level. CVD, cardiovascular disease.

## Discussion

Levels of total plasma triglycerides and cholesterol, as well as LDL and HDL, have traditionally been monitored as predictors of cardiovascular events [31], [32]. In our study, we have investigated beyond total LDL levels and have examined whether plasma levels of electronegative L5 LDL may be a novel predictor of CVD. Similar to our previous findings, we showed that plasma L5 levels are increased in asymptomatic individuals with MetS [26]. Furthermore, we found that plasma L5 levels in asymptomatic MetS subjects were not only correlated with CVD risk factors and CVD risk but were also correlated with the number of MetS criteria fulfilled and, therefore, CVD progression. These strong correlations demonstrate that circulating L5 may have potential as a predictor of CVD progression and adverse cardiac events.

Several metabolic risk factors tend to cluster in middle-aged adults, including increased BMI and SBP, in addition to elevated levels of LDL, total cholesterol, triglycerides, and blood glucose. Each criterion of MetS is related to an increased risk of developing CVD, and some risk factors are related to a greater risk of coronary heart disease. In terms of attributable deaths, the leading CVD risk factor is elevated blood pressure (to which 13% of global deaths are attributed), followed by tobacco use (9%), elevated blood glucose (6%), physical inactivity (6%), and overweight/obesity (5%) [34]. Men with MetS have been shown to be 2.9 (95% confidence interval [CI], 1.2–7.2) to 4.2 (95% CI, 1.6–10.8) times more likely to die of coronary heart disease after adjusting for conventional cardiovascular risk factors [35]. Although the majority of CVD is caused by risk factors that can be controlled, treated, or modified [3]–[5], some major CVD risk factors may be unknown and thus cannot be controlled.

Both LDL (–) and L5 are naturally occurring forms of LDL [9], [14]. LDL (–) can induce vascular endothelial cells and monocytes to release multiple cytokines, including interleukin-8, monocyte chemoattractant protein-1, tumor necrosis factor-α, and vascular cell adhesion molecule-1 [11], [36]–[39]. Furthermore, L5 can induce endothelial cell apoptosis [14] and cardiomyocyte apoptosis indirectly through endothelial cell–released chemokines [38], which are both critical events in the development of atherothrombosis and CVD [40], [41]. Thus, it has been suggested that elevated L5 levels may induce the development of atherothrombosis and CVD. In the current study, we showed that L5 but not L1 induced a marked increase in cell apoptosis and morphological changes in primary BAECs, which is consistent with previously reported findings [20]. L5 from non-MetS control subjects was as cytotoxic as L5 from MetS patients (both at a concentration of 50 µg/mL), indicating that increased concentrations of L5 in patients with MetS may contribute increased cytotoxicity in these individuals. The pathogenic role of L5 supports our present findings, indicating that L5 may be useful a predictor of CVD.

Modified LDL particles have been reported to play key roles in all stages of atherosclerosis. Oxidized LDL (oxLDL) and minimally modified LDL (mmLDL), a mildly oxidized LDL, are the most widely studied types of modified LDL [42]. Because LDL(–) and L5 are each pools of LDL particles modified by several mechanisms, oxLDL would account only for a variable small proportion of their composition [9], [15]. The increased electronegativity and negative charge of these LDL particles are attributed to other non-oxidative causes [43]. In vitro and invivo studies have shown that oxLDL promotes endothelial cell toxicity and vasoconstriction [44], and oxLDL has been reported to be a predictor of secondary cardiovascular events. In a prospective study, baseline oxLDL levels were an independent predictor of cardiac death, nonfatal myocardial infarction, and unstable angina; patients in the highest quartile had a hazard ratio 3.15 times greater than did patients in the lowest quartile [45]. In addition, MetS subjects have higher levels of oxLDL than control subjects [46], although the number of MetS criteria was not shown to be associated with oxLDL levels [47]. Because the level of oxLDL circulating in the blood is very low, it is difficult to demonstrate its association with CVD risk factors; thus, the relationship between oxLDL and CVD risk factors has not been fully established [42]. Therefore, oxLDL may not be as useful as a biomarker in asymptomatic individuals with CVD.

Age is a CVD risk factor, as observed in previous studies [31], [32]. In the present study, the mean age of MetS subjects was higher than that of control subjects (Table 1). However, the Spearman rank correlation coefficient revealed no association between age and L5% (Table 2). Thus, the L5 differential between the control and MetS groups can be attributed to the pathology of CVD progression rather than the age difference between these groups, suggesting that L5 could potentially be considered a marker of CVD pathology.

The major limitation of this study was the cross-sectional study design, which was used because this was a discovery study. Although we are confident that our findings show an association between L5% and the progression of CVD, we cannot conclude from our data whether L5 plays a role in the causality of CVD. In addition, because we used questionnaires in our study, a Neyman bias may have affected our results. Although we believe our questionnaire was objectively and well written, the possibility remains that subject answers may have lacked accuracy, in turn minimizing or maximizing the effects of certain variables. Finally, because we did not obtain prospective follow-up data from these patients regarding CVD events, we estimated CVD risk according to the Framingham risk score.

In summary, we showed that L5% and [L5] was associated with the degree of MetS in asymptomatic individuals. Furthermore, L5% correlated strongly with various CVD risk factors, and fasting plasma glucose level and BMI explained 28% of the variation in L5%. Moreover, L5% was associated with CVD risk and contributed to 11% of the 30-year general CVD risk variance when controlling for waist circumference. Our findings indicate that LDL electronegativity was associated with multiple CVD risk factors and CVD risk, suggesting that the LDL electronegativity index may have the potential to be a novel index for predicting CVD. Future prospective studies will aim to identify a causal relationship between L5% and the progression of CVD and adverse cardiac events.
